# Exploring Clinician Perspectives on Artificial Intelligence in Primary Care: Qualitative Systematic Review and Meta-Synthesis

**DOI:** 10.2196/72210

**Published:** 2026-02-05

**Authors:** Robin Bogdanffy, Alisa Mundzic, Peter Nymberg, David Sundemo, Anna Moberg, Carl Wikberg, Ronny Kent Gunnarsson, Jonathan Widén, Pär-Daniel Sundvall, Artin Entezarjou

**Affiliations:** 1 General Practice/Family Medicine, School of Public Health and Community Medicine, Institute of Medicine, Sahlgrenska Academy, University of Gothenburg, Huvudbyggnad Vasaparken, Universitetsplatsen 1, Gothenburg, 40530, Sweden, 46 317860000; 2 Center for Primary Health Care Research, Department of Clinical Sciences, Malmö, Lund University, Malmö, Sweden; 3 University Clinic Primary Care Skåne, Region Skåne, Malmö, Sweden; 4 Center for Digital Health, Sahlgrenska University Hospital, Region Västra Götaland, Mölndal, Sweden; 5 Department of Health, Medicine and Caring Sciences, Faculty of Medicine and Health Sciences, Linköping University, Linköping, Sweden; 6 Research, Education, Development & Innovation, Primary Health Care, Region Vastra Gotaland, Gothenburg, Sweden; 7 College of Medicine and Dentistry, James Cook University, Cairns, Australia

**Keywords:** artificial intelligence, large language models, natural language processing, generative artificial intelligence, primary health care, attitude of health personnel, systematic reviews as topic, qualitative research.

## Abstract

**Background:**

Recent advances have highlighted the potential of artificial intelligence (AI) systems to assist clinicians with administrative and clinical tasks, but concerns regarding biases, lack of regulation, and potential technical issues pose significant challenges. The lack of a clear definition of AI, combined with limited focus on qualitative research exploring clinicians' perspectives, has limited the understanding of perspectives on AI in primary health care settings.

**Objective:**

This review aims to synthesize current qualitative research on the perspectives of clinicians on AI in primary care settings.

**Methods:**

A systematic search was conducted in MEDLINE (PubMed), Scopus, Web of Science, and CINAHL (EBSCOhost) databases for publications from inception to February 5, 2024. The search strategy was designed using the Sample, Phenomenon of Interest, Design, Evaluation, and Research type (SPIDER) framework. Studies were eligible if they were published in English, peer-reviewed, and provided qualitative analyses of clinician perspectives on AI in primary health care. Studies were excluded if they were gray literature, used questionnaires, surveys, or similar methods for data collection, or if the perspectives of clinicians were not distinguishable from those of nonclinicians. A qualitative systematic review and thematic synthesis were performed. The Grading of Recommendations Assessment, Development and Evaluation-Confidence in Evidence from Reviews of Qualitative Research (GRADE-CERQual) approach was used to assess confidence in the findings. The CASP (Critical Appraisal Skills Program) checklist for qualitative research was used for risk-of-bias and quality appraisal.

**Results:**

A total of 1492 records were identified, of which 13 studies from 6 countries were included, representing qualitative data from 238 primary care physicians, nurses, physiotherapists, and other health care professionals providing direct patient care. Eight descriptive themes were identified and synthesized into 3 analytical themes using thematic synthesis: (1) the human-machine relationship, describing clinicians’ thoughts on AI assistance in administration and clinical work, interactions between clinicians, patients, and AI, and resistance and skepticism toward AI; (2) the technologically enhanced clinic, highlighting the effects of AI on the workplace, fear of errors, and desired features; and (3) the societal impact of AI, reflecting concerns about data privacy, medicolegal liability, and bias. GRADE-CERQual assessment rated confidence as high in 15 findings, moderate in 5 findings, and low in 1 finding.

**Conclusions:**

Clinicians view AI as a technology that can both enhance and complicate primary health care. While AI can provide substantial support, its integration into health care requires careful consideration of ethical implications, technical reliability, and the maintenance of human oversight. Interpretation is constrained by heterogeneity in qualitative methods and the diversity of AI technologies examined across studies. More in-depth qualitative research on the effects of AI on clinicians’ careers and autonomy could prove helpful for the future development of AI systems.

## Introduction

### Background

Health care systems worldwide are increasingly strained, partly due to aging populations and insufficient resources, and there is increased demand for accessibility, medical quality, and economic efficiency [1]. Primary care is regarded as a cornerstone in health care systems across many regions of the world [2], and primary care clinicians’ job satisfaction is considered essential for many health care systems [3]. Recent studies have demonstrated the potential of artificial intelligence (AI) tools and systems to reduce burnout and increase the efficiency of health care professionals [4], as well as to improve diagnostic accuracy and patient care [5].

AI is an emerging technology with a broad range of applications [6-8]. However, there is still no consensus on a general definition of AI, which presents an obstacle to investigating peoples’ perspectives [9].

Recent advances in AI have led to increased health care–related AI use and research [10]. Previous reports have indicated that the main applications of AI in primary health care have been data extraction and processing [11], reducing administrative burden [12], and assisting physicians in diagnosing, determining a prognosis, and choosing a treatment [13]. Current large language models (LLMs) have started to play a more prominent role in health care, and new applications are frequently identified [14]. Several LLM products, including Chat Generative Pretrained Transformer (GPT), have demonstrated the capability of medical reasoning and have performed well on medical licensing exams [15,16]. Moreover, LLMs may improve communication between health care professionals and patients through text simplification [17].

Previous research suggests concerns among clinicians regarding the use of AI in health care, such as demographic biases, insufficient regulation, lack of trust in AI systems [18], and automation bias [19].

While there seems to be a lack of systematic synthesis on clinicians’ perspectives on AI in primary health care, a scoping review conducted in 2022 on perceptions and needs of AI in health care identified few studies within primary health care. End-user and stakeholder opinions are essential for future implementation and development. Since research on AI in primary care is limited and results are varied, perceptions of the use of AI in this domain are not fully understood [7].

### Definitions

#### Definition of Clinicians

In this review, we refer to health care professionals who provide direct patient care (eg, physicians, nurses, physiotherapists) as clinicians.

#### AI Definitions

**Figure 1.**
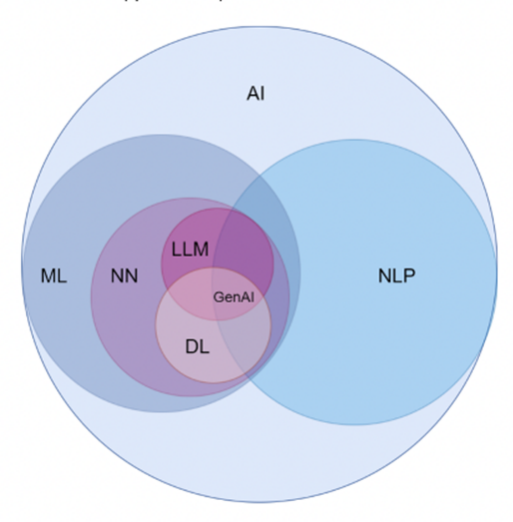
Conceptual hierarchy of AI domains. AI: artificial intelligence; DL: deep learning; GenAI: generative artificial intelligence; LLM: large language model; ML: machine learning; NLP: natural language processing; NN: neural network.

Different AI systems vary in their levels of autonomy and adaptiveness after deployment [20]. For broad inclusion, this review included any AI system or concept specified by the study authors as AI. This includes LLMs, generative AI (GAI), natural language processing (NLP), and clinical decision support systems (CDSS). The definitions of these model types are complex, and overlap exists; LLMs are language models trained on large amounts of data and are created to process and generate human language based on prompts created by the user, sometimes operating as GAI or as the core of a CDSS [21]. GAI refers to AI which is capable of generating content, such as text, images, or audio, some of which are based on LLMs. Current GAI system examples are GPT-4, Copilot, and DALL-E 2 [22]. The term NLP encompasses computational techniques designed for the automatic analysis and representation of language [23]. A CDSS is an information system that generates specific clinical recommendations through certain software-based algorithms [24]. An illustration of key concepts of AI and machine learning (ML) is provided in Figure 1 [25].

### Objective

The aim of this systematic review is to synthesize the current qualitative research on clinicians’ perspectives on AI in primary care settings.

## Methods

### Study Design

We performed a systematic review and metasynthesis in accordance with the Cochrane Qualitative and Implementation Methods Group [26]. The review was reported according to the Enhanced Transparency in Reporting the Synthesis of Qualitative Research (ENTREQ) statement [27] (Checklist 1) and Preferred Reporting Items for Systematic Reviews and Meta-Analyses (PRISMA) guidelines [28] (Checklist 2). The study protocol was registered with the International Prospective Register for Systematic Reviews (PROSPERO; CRD42024505209) [29] before conducting the review. This study employed a methodology for a systematic review of qualitative studies, in which the authors conducted a secondary qualitative synthesis of published clinician quotes and primary authors’ interpretations from the reviewed studies, allowing for deeper exploration of underlying patterns and themes.

### Search Strategy

#### Overview

The search strategy was developed using the Sample, Phenomenon of Interest, Design, Evaluation, and Research type (SPIDER) framework [30]: clinicians in primary care (Sample); their perspectives and experiences regarding AI (Phenomenon of Interest); explored through qualitative study designs (Design); focusing on evaluations of experiences, attitudes, perspectives, and views (Evaluation), within qualitative and mixed methods research (Research type). Search strings were designed by the author team and reviewed by a health sciences librarian at the Gothenburg University Library. Broader terms for “primary health care,” “artificial intelligence,” and “perspectives” were combined. Controlled vocabulary and free-text terms were used (Multimedia Appendix 1). A systematic search was conducted in MEDLINE (PubMed), Scopus, Web of Science, and CINAHL (EBSCOhost) databases for publications from inception to February 5, 2024. Backward citation searching of the reference lists of the included articles was also performed. Search strings were modified according to the requirements of each database. All searches were performed independently by authors RB and AM and reviewed by author AE. Search documentation is presented in accordance with the Preferred Reporting Items for Systematic Reviews and Meta-Analyses Search (PRISMA-S) checklist [31] (Checklist 3).

#### Inclusion Criteria

Studies were included if they were conducted in a primary health care setting, involved clinicians such as doctors, nurses, physiotherapists, or other health care professionals providing direct patient care, and explored any perspectives on AI in primary health care. For the purposes of this review, studies were considered to be conducted in a primary health care setting if participants were recruited via primary care services, had documented interaction with primary care, or if the study context clearly reflected a primary care environment such as general practice or family medicine. Only qualitative and mixed methods studies published in English in peer-reviewed scientific journals were eligible for inclusion.

#### Exclusion Criteria

Studies were excluded if they lacked sufficient qualitative depth, such as those using only questionnaires, surveys, or similar methods for data collection. We also excluded studies in which qualitative data on clinicians’ perspectives were not clearly distinguishable from those of nonclinicians, as well as grey literature and unpublished materials.

### Study Selection

Authors RB and AM imported the search results into Rayyan (citation manager) [32], where duplicates were removed. The authors independently screened titles and abstracts of the remaining articles against the inclusion and exclusion criteria. Any disagreements were discussed, and if consensus was not reached, a third author (AE) was consulted for a final decision. We included articles claiming to evaluate AI technology based on the authors’ definition of AI, as described in the “Introduction.”

### Critical Appraisal

Authors RB and AM independently conducted critical appraisal using the Critical Appraisal Skills Program (CASP) checklist for qualitative research [33]. Disagreements were discussed until a consensus was reached or author AE was consulted for a final decision.

### Data Analysis and Synthesis

Data were extracted from the Results section of the included articles and their supplementary material. Participant quotes and authors’ findings were analyzed independently by RB and AM to generate descriptive themes using thematic analysis according to the Braun and Clarke method [34]. This involved several steps through a primarily inductive analytic process. First, the authors familiarized themselves with the extracted data by reading it several times. RB then developed codes using line-by-line coding of words or sentences considered meaningful, using the NVivo software [35]. Data extraction and coding were performed in 2 stages. The first stage involved articles solely containing primary care clinician perspectives, and the second stage involved articles containing perspectives of both primary care clinicians and nonclinical health care professionals. Qualitative data with perspectives other than that of clinicians was not coded. Codes were discussed by both authors until an agreement was reached, whereafter, they were exported to a Microsoft Excel spreadsheet. RB then proceeded to generate descriptive themes by grouping codes. The alignment of codes to certain themes was discussed, and the descriptive themes were refined. Thematic synthesis, according to the Thomas and Harden method, was employed to develop higher-order analytical themes. It is a well-suited method for exploring qualitative data such as perspectives or sentiments [36]. Thematic synthesis was accomplished through a discussion between both authors, during which the analytical themes were developed and named (Multimedia Appendix 2). No new themes emerged from coding the articles with mixed perspectives. This method was chosen due to its ability to identify recurring themes and patterns across multiple studies, enhancing the breadth of the analysis.

### Assessment of Confidence in the Evidence

Confidence in each synthesized finding was assessed using the Grading of Recommendations Assessment, Development and Evaluation-Confidence in Evidence from Reviews of Qualitative Research (GRADE-CERQual) approach. The GRADE-CERQual approach was chosen as it explicitly addresses qualitative evidence synthesis, allowing systematic and transparent assessments of the confidence in each thematic finding. Authors RB and AM independently evaluated each finding based on 4 components: methodological limitations, coherence, adequacy, and relevance. Each component was assessed as having no or very minor, minor, moderate, or serious concerns. Discrepancies were discussed, and if agreement was not reached, author AE was consulted for a final decision. Each finding began with an initial rating of “high confidence”. Confidence levels were then potentially downgraded to moderate, low, or very low based on the severity and number of concerns present in each component. Typically, one level of downgrading (eg, from high to moderate confidence) was applied when moderate concerns were identified in one component combined with minor concerns in other components, and two levels (eg, from high to low confidence) were applied when serious concerns or multiple moderate concerns were present. When concerns were minor or very minor, no downgrading was performed [37-42].

### Ethical Considerations

Because this systematic review used only pre-existing data, ethical approval was not required.

## Results

### Search Results and Selection

**Figure 2.**
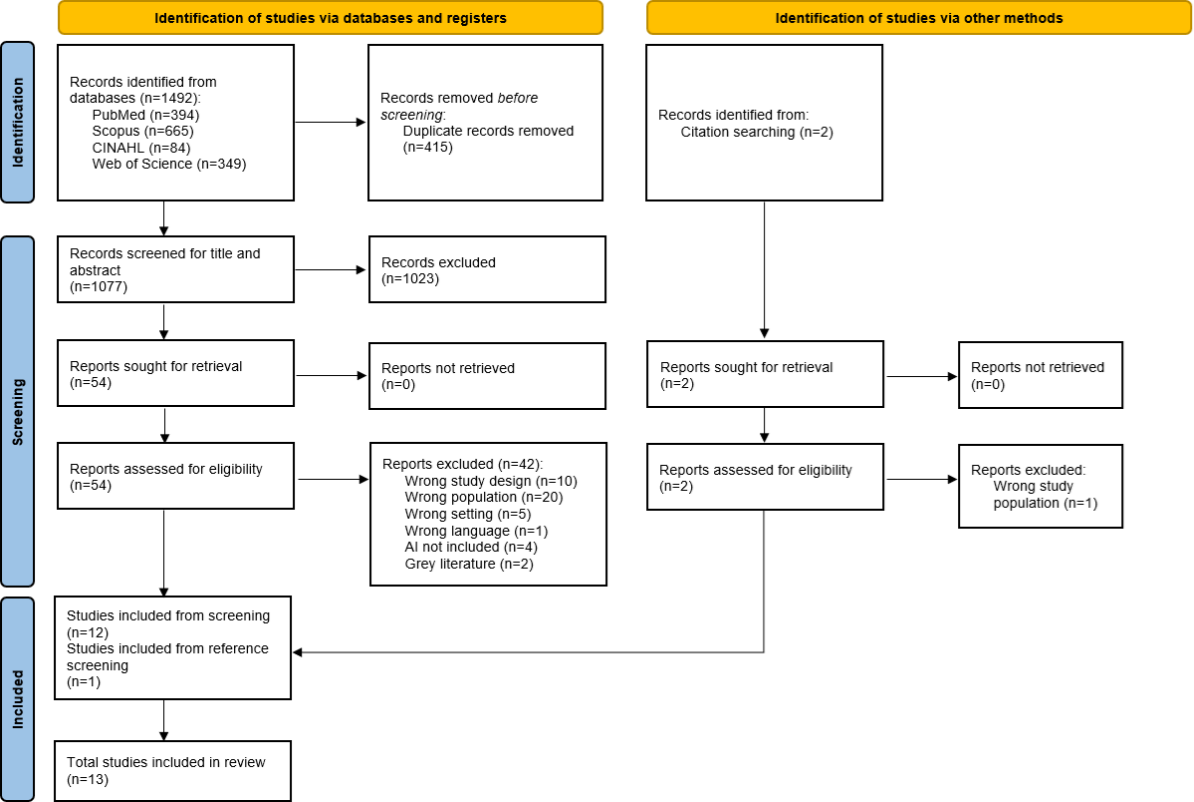
Preferred Reporting Items for Systematic Reviews and Meta-Analyses (PRISMA) flow diagram of study selection.

The final search generated 1492 results, and 415/1492 (27.8%) duplicates were excluded. The remaining 1077/1492 (72.2%) articles were screened by title and abstract, and 54/1077 (5%) articles were retrieved in full text and evaluated, of which 42/54 (77.8%) were excluded based on the exclusion criteria. Finally, 12/54 (22.2%) articles were included from the screening, and 1 additional article was identified from the reference lists of the previously included articles, resulting in a total of 13 studies [43-55] (Figure 2).

The 13 included studies were conducted in 6 different countries. Australia was the most frequent location with 4/13 (30.8%) studies [45-47,49], followed by Canada with 3/13 (23.1%) studies [51-53] and the United States with 3/13 (23.1%) studies [43,44,50]. Sweden [55], the Netherlands [54], and Germany [48] each contributed 1/13 (7.7%) study. Many studies used semistructured interviews for data collection (6/13, 46.2%) [43,45,48,51,53,55]. Mixed methods were used in 3/13 (23.1%) studies [44,47,50], focus groups in 2/13 (15.4%) studies [49,54], deliberative dialogue in 1/13 (7.7%) study [52], and a co-design workshop in 1/13 (7.7%) study [46]. Characteristics of the included studies are presented in Table 1.

**Table 1.** Characteristics of the included studies.

Study	Country	Method	Characteristics of participants	Occupation	Type of AI	Identified themes
Davis et al [43]	USA	Semistructured interviews	n=10 Age^a^ Gender^a^	Mixed^b^	Machine learning	Acceptability, Clinical Utility, Privacy, Data and Evidence, Clarification/Confusion, Communication, Patient, Family and Provider Characteristics and Experiences, Inner Setting, Outer Setting, Suggestions
Litvin et al [44]	USA	Mixed methods	n=39 Age^a^ Gender^a^	Mixed^b^	CDSS^c^	Provider factors related to CDSS^c^ adoption, Patient factors related to CDSS adoption, Technical factors related to CDSS adoption, Organizational factors related to CDSS adoption
Navarro et al [45]	Australia	Semistructured interviews	n=10 Age^a^ Gender-men: n=7, women: n=3	General practitioners	Natural language processing	Doctor-AI^d^ collaboration, Desired features, Concerns and challenges, Consultation of the future
Kocaballi et al [46]	Australia	Co-design workshop	n=16 Age^a^ Gender-men: n=10, women: n=6	General practitioners	Generative AI	Professional autonomy, Human-AI collaboration, New models of care
Shibl et al [47]	Australia	Mixed methods	n=37 Age^a^ Gender-men: n=24, women: n=13	General practitioners	CDSS	Usefulness, Facilitating conditions, Ease of use, Social influence, Trust in the knowledge base, Involvement, Moderating variables
Buck et al [48]	Germany	Semistructured interviews	n=18 Age (y): 34-70 Gender-men: n=9, women: n=9	General practitioners	CDSS	Concerns, Expectations, Environmental influences, Individual characteristics, Minimum requirements of AI-enabled systems
Ahearn et al [49]	Australia	Focus groups	n=22 Gender-men: n=15, women: n=7	General practitioners	CDSS	Reaction to prompts, Concerns and potential problems, Effects on prescribing behavior, Need for training, Helpful features of decision support systems, Suggested improvements, Attitudes to evidence-based guidelines
Allen et al [50]	USA	Mixed methods	n=15 Age^a^ Gender^a^	General practitioners	Unspecified AI	Concerns regarding technology, Concerns regarding people and processes
Nash et al [51]	Canada	Semistructured interviews	n=10 Age^a^ Gender^a^	Mixed^b^	Unspecified AI	Context of Health Care Setting, Knowledge, Foundation of Trust: Accuracy, Experience, and Openness, Internal and External Influences, Anticipated Impact of AI
Upshaw et al [52]	Canada	Deliberative dialogue	n=21 Age (y): 28-64 Gender-men: n=12, women: n=9	Mixed^b^	CDSS	Priority applications of AI in primary care, Impact of AI on primary care provider roles, Considerations for provider training in AI
Libon et al [53]	Canada	Semistructured interviews	n=8 Age^a^ Gender^a^	Mixed^b^	Unspecified AI	Provider satisfaction, Difficulties with implementation, Impact on patient care
Sangers et al [54]	Netherlands	Focus groups	n=17 Age (y): 31-62 Gender-men: n=7, women: n=10	General practitioners	Unspecified AI	Perceived Benefits, Perceived Barriers, Preconditions for Implementation
Helenason et al [55]	Sweden	Semistructured interviews	n=15 Age^a^ Gender^a^	General practitioners	CDSS	Trust, Usability and User Experience, Clinical Context

a Age/Gender: undisclosed or not distinguishable from nonclinicians.

b Mixed: doctors, nurses, physiotherapists, or other health care professionals providing direct patient care.

c CDSS: clinical decision support system.

d AI: artificial intelligence.

### Critical Appraisal

The critical appraisal using the CASP qualitative checklist indicated that all included studies had clear research aims, appropriate qualitative methodologies, and well-reported findings. Several studies lacked sufficient transparency in ethical considerations (Multimedia Appendix 3).

### Findings

The thematic analysis identified eight descriptive themes. These descriptive themes represent recurring elements identified across studies and served as the foundation for the synthesis of broader analytical themes. Thematic synthesis resulted in three analytical themes: the human-machine relationship, the technologically enhanced clinic, and the societal impact of AI. All themes are presented in Table 2. There was a wide time span across the included studies. Earlier studies, conducted between 2003 and 2013, explored perspectives on less advanced systems, such as CDSSs not based on ML [44,47,49], whereas later studies included more advanced AI systems, such as GAI and NLP [43,45,46,48,50-54], or a CDSS based on ML [55].

**Table 2.** Analytical themes and descriptive themes.

The human-machine relationship	The technologically enhanced clinic	The societal impact of AI^a^
Interaction with AI	Workplace changes	Bias
Resistance to AI	Technological concerns	Data security, privacy, and legal implications
	Clinical impact	
	Desired features	

a AI: artificial intelligence.

### Theme 1: The Human-Machine Relationship

#### Interaction With AI

The relationship between humans and AI was approached from different perspectives, and several clinicians perceive AI as an assistant that could alleviate the burden of specific tasks [45-47,51-55]. Some clinicians suggested that AI may have a negative impact on the clinician-patient relationship through the lack of a human connection [46,48]. However, some findings suggested increased clinician empathy with AI assistance [46,50] or even facilitation of communication between a clinician and a patient [45]. Several clinicians wished for AI to enhance rather than replace the relationship between clinician and patient [52]. The idea of working with the AI algorithm to present information and decisions to the patient was also appreciated [43]. It was also believed that the use of AI could increase time spent with patients rather than other tasks [45]:

*Yes, just taking my hands off the computer, getting my eyes off the screen, so that I can be spending time with the patient. And also saving me the documentation time, because you can either spend more time with the patient or see more patients*.

[GP]

Some clinicians believed that a patient’s confidence in the clinician would increase by using AI [53,55], and others believed that AI would empower clinicians to be more confident in their practice [53-55]. Several clinicians thought that AI could be valuable in educating clinicians or providing new clinical insights [51,53,54]. However, there were concerns that by introducing AI systems to inexperienced clinicians, there could be a risk of declining proficiency due to the automation of tasks [52]. Clinicians highlighted that AI could complement human medical practitioners with nonhuman traits, such as the ability to not get tired, thus retaining its clinical accuracy [48]. Many clinicians believed that AI could improve the clinical consultation by shifting the clinician’s focus toward the patient [45,46,52]. Clinicians in one study believed that AI systems currently focus more on task efficiency than on improving patient care [46].

Some clinicians ultimately wished to retain control over the AI system, keeping the clinician in charge [45,46,55]. Whether clinicians wanted to have a deeper understanding of the programming behind an AI system differed, with some clinicians having a desire for a more profound knowledge [48] and others believing that it was not required [43,50]. Clinicians’ trust in AI systems was discussed with conflicting opinions, where some believed that AI could ultimately be trusted, and some did not [45,47,55]. Trust in the AI system would increase if it were scientifically proven to work or validated by other health care professionals, according to some clinicians [55]. The topic was further investigated in discussions surrounding trust in the AI creators, where clinicians expressed that they would trust the system if it were based on a well-known physician or author. They voiced no concern regarding how the system was developed or who the software developers were [47].

#### Resistance to AI

Several clinicians voiced concerns regarding AI replacing medical staff or jobs in other sectors [46,48,50]. Some expecting doctors to eventually assist AI [46]:

*I think eventually the doctors will be the assistant doctors … Doctors will assist artificial intelligence what to do … eventually … we'll be helping it. I think we'll be assistant … Because they'll be doing everything. It will be just saying, yes, no, yes, no. Say supervision, but we'll be assisting*.

[GP]

Other clinicians dismissed such fears [45,52]. It was also thought that clinicians’ gut feelings could not be replaced by AI [51]. There was also resistance or skepticism toward AI systems. Several clinicians voiced potential negative effects on their workflows, stating that they perceived AI to cause increased time expenditure [43,47,48,50]. Other clinicians believed that there was no change in time expenditure [44]. It was also believed that decreased time expenditure could have adverse effects due to patients becoming accustomed to the increased speed and effectiveness of certain processes [50].

Previous negative experiences with the introduction of electronic health records could influence skepticism toward AI [51,52]. Discussions about factors contributing to AI resistance emerged, and some clinicians concluded that this could be caused by age, personal interests, or alignment with accepting new technology [45,48,50]. Other barriers, such as being limited by time or resources, were also mentioned [43]. Some clinicians were worried about patient safety due to concerns about AI safety and algorithmic bias [52].

### Theme 2: The Technologically Enhanced Clinic

#### Workplace Changes

There were different clinician perspectives on automating certain tasks, the impact on workload, and integrating a new system in a workplace [43-55]. Clinicians expressed a belief that using AI systems could potentially save time through automation of administrative tasks or clinical decision support [44-48,50,54,55]. Some voiced that this was the foremost reason for using AI [45]:

*I'd be confident that it would save me time but not replace me thinking, which is not the aim, for me it’s the saving time*.

[GP]

Not all clinicians agreed on this topic. Some thought that AI would increase their workload by complicating their tasks [43,50] or disrupting their workflow and disturbing their train of thought [47,53]. Interference with the clinician’s decision-making process by the suggestion of unnecessary tests was highlighted as a negative impact on workflow [43]. Opinions were mixed regarding whether cost was an important factor for implementation. Some believed that cost could be a factor in system acceptance [48], whereas others did not [47]. Some thought that there was probably a positive cost-benefit for clinics using AI systems [54]. Some clinicians wished that the focus of AI systems should be to assist patient care and not strictly for financial gain [47]. Assisting clinicians in primary care centers was thought especially important in countering physician burnout [51]. Several studies voiced concerns regarding integrating AI systems into already established working environments. Many clinicians wished for seamless integration of AI systems into existing systems [45,47-50,52], whereas some wished for the systems to be completely separated [49,55]. The need for established policies and routines prior to AI system adoption was also voiced [55].

#### Technological Concerns

Clinicians voiced several technological concerns, including the risk of technical issues, issues with the AI system itself, or user errors [43-52,55]. Several clinicians had experience with CDSS providing inaccurate information [47] or leaving out important information [49]. Many clinicians were concerned with the risk of AI producing erroneous information or having a low accuracy [45-47,49,50,55]. The AI’s ability to decide whether a piece of information was relevant was also a concern [45].

Simultaneously, there was also a fear of user error, meaning clinicians were uncomfortable using the system and potentially causing errors [45,47,51]. Several clinicians further explored this subject, mentioning that they would fear not knowing how to use the system in front of patients [47]. The reason for this was not further specified, but other clinicians deemed using an AI system nonintuitive [44]. Another technical concern expressed by some clinicians was the possibility of complete system failure [48]:

*If my system goes down, my AI is on standby, then sorry, I can’t diagnose, my system strikes out. That is why it’s nice to be able to write down with a pen on paper what a patient has and has received*.

[GP]

Accessibility was approached from different perspectives. There were wishes for AI systems to be easily accessible from the electronic health record [43]. Clinicians also expressed concern that some patient groups could be less likely to have access to the technology needed for AI interaction [43]. Likewise, using AI as triage could be inaccessible for some populations [52]. Computer and AI training for clinicians was generally seen as an important factor for implementation.

Clinicians from different studies expressed the need for specific training [47,52,55] and being regularly informed about AI technology [48]. Some believed there was no need for training, as they had been using an AI system without prior professional training [47]. The growing use of similar algorithms or programs, such as CDSS or other AI systems, was generally considered beneficial for implementation [43]. Even though several technical concerns were voiced, remarks from one study were that technological advancements in medicine are also necessary. It would help clinicians stay up-to-date with the increasing amount of medical knowledge, enable predictive models, and keep up with demographic changes, making clinics technologically modern for younger physicians [48].

#### Clinical Impact

Thoughts on how the usage of AI would impact clinical work emerged in multiple studies [44-49,53-55]. Some mentioned the benefits of diagnostic support, increasing clinical effectiveness and accuracy [44,45,47-49,53-55], while others mentioned positive effects on their prescribing behavior by using a pharmaceutical decision support system [49]. Using AI technology in remote diagnostics or examinations was considered beneficial [53]. Clinicians also discussed retrieving a medical history using AI as a helpful tool [45]. However, some clinicians thought that by removing the act of writing from the clinician, their thought process could be disrupted [46]:

*One of the advantages of when you write it is it reinforces what you thought … It’s a thinking process, because you actually think about what this actually means? … How can you capture that writing experience in an electronic medium?*

[GP]

#### Desired Features

Clinicians had several ideas regarding features they would like to see in AI systems and decision support systems. Their wishes for specific features and the implications of these features were recurring topics [44-52,55]. Many expressed the importance of the extraction and summarization of essential data [45,52]. They also expected AI systems to process more information than any human brain could, all while maintaining a high working speed [48]. Clinicians wished for AI to be more accurate and yield better results than humans so that it would not be considered obsolete [48,51]. The possibility for clinicians to customize the information presented or for the AI to adapt to the clinicians’ needs was deemed important [44,46,49,50].

Other desired features were AI-assisted patient triage [48,52], identifying patients with high risk of disease [52] and integrating AI into telehealth systems [46,48,52]. Besides purely clinical functions, suggestions included using AI to predict visit surges and for health resource planning [52]. Numerous clinicians emphasized the necessity for AI systems to be user-friendly, thereby enhancing the probability of their adoption [47-49,51,55].

Some clinicians felt strongly that the AI should provide a clear, logical explanation of how it arrived at its conclusions, emphasizing the need for transparency and traceability of the AI’s algorithm. Others, however, argued that as long as the AI’s output is accurate and reliable, understanding its inner workings is less important [50]. Some clinicians also expressed that their limited knowledge of AI systems hindered them from providing ideas on possible features [51]:

*And I do not know enough about artificial intelligence to give you big ideas of what could be done*.

[Health care provider]

### Theme 3: The Societal Impact of AI

#### Bias

Clinicians discussed several types of bias, some of which could affect the population and others which might affect clinicians. Opinions regarding bias and how it could affect our society were expressed [45,46,48,50-52]. Clinicians were worried that the data used to train an AI system could be historically biased by being trained mainly on information from only one or a few demographic groups [46]. They also expressed that bias could “leak” into the AI from its creators [50]:

*The thing I’m apprehensive about is, how are we teaching AI these things because some of those biases could leak in*.

[GP]

Clinicians were additionally concerned that automation bias could affect the clinician’s decision-making or potentially distract the clinician from important information. This means that clinicians could over-rely on the information presented by AI and prioritize it over their own reasoning [45,46,48,51,52].

#### Data Security, Privacy, and Legal Implications

Patient data security and privacy were topics discussed from different perspectives. Clinicians expressed concerns about the risks of having sensitive data processed through AI and the impact it could have both on patients and clinicians [43,45-48,50]. Many clinicians voiced concern over the security surrounding data processing, whether the data would be encrypted, and the risks of hacking or misuse of data [45-48]. On the other hand, some clinicians did not consider security important since they were unaware of any security issues [47].

Other clinicians wanted to know how the AI system handled data privacy [45]. Some clinicians thought that letting AI systems process all the data produced in a clinical setting could be an inherent problem, leading to possible monitoring of clinicians. However, none of the participants could identify who would benefit from such surveillance [48]. Some clinicians took a negative stance on the complete transparency potentially caused by using AI in documentation, as opposed to the natural filtration of information employed by clinicians. They also preferred keeping data from a patient-physician consultation private [48]:

*Patient data are very sensitive data. Disease data are very sensitive data. [There is the risk that] they are passed on somewhere, that some authorities who have nothing to do with it or should have nothing to do with it could intercept the data and use this to the disadvantage of the patients*.

[GP]

In addition to patient data safety, the safety of doctors and how the availability and transparency of data could work against them were considered. Clinicians suggested that doctor safety could be jeopardized when using AI for documentation or decision support. An example given by clinicians was if the AI had suggested something that a clinician did not take notice of or if there could be compromising data in what the AI system documented [46].

Legal implications were also a large topic of discussion in several studies. Many clinicians voiced legal concerns, primarily related to fear of legal action taken toward the clinician if they acted outside of recommendations given by the AI system [45,46,50,55]. Another viewpoint was that clinicians expected there to be built-in legal protection that shifted responsibility from the clinician to an AI system [48], or for there to be a clearly defined medicolegal liability [52,55].

### Assessment of Confidence

The results of the GRADE-CERQual assessment for the review findings are summarized in Table 3.

**Table 3.** Summary of qualitative findings, Grading of Recommendations Assessment, Development and Evaluation-Confidence in Evidence from Reviews of Qualitative Research (GRADE-CERQual) assessments.

Summary of review findings	References	CERQual^a^ assessment of confidence in the evidence	Explanation of CERQual assessment
Interaction with AI^b^			
Clinicians perceive AI as an assistant that could alleviate the burden of specific tasks.	[45-47,51-55]	High confidence	There were minor concerns regarding adequacy.
AI may negatively impact the clinician-patient relationship due to a lack of human connection.	[46,48]	Low confidence	There were serious concerns regarding adequacy, moderate concerns regarding coherence, and minor concerns regarding methodology, justifying two levels of confidence downgrade.
AI could enhance clinician empathy or facilitate clinician-patient communication and confidence.	[43,45,46,50,52-55]	Moderate confidence	There were moderate concerns regarding coherence and minor concerns regarding adequacy, justifying one level of confidence downgrade.
Clinicians wish to retain control over AI systems and understand how they function.	[43,45,46,48,50,55]	High confidence	There were moderate concerns regarding adequacy, and minor concerns regarding methodology and coherence.
Clinicians expressed conflicting views regarding trust in AI.	[45,47,55]	High confidence	There were moderate concerns regarding adequacy. The concern was not deemed serious enough for a downgrade of confidence.
Resistance to AI			
Some clinicians fear being replaced or having their role diminished by AI.	[46,48,50]	Moderate confidence	There were moderate concerns regarding adequacy, and minor concerns regarding methodology and coherence, justifying one level of confidence downgrade.
The introduction of AI could increase clinicians’ time expenditure or disrupt workflows.	[43,44,47,48,50]	Moderate confidence	There were moderate concerns regarding coherence and minor concerns regarding methodology and adequacy, justifying one level of confidence downgrade.
Multiple factors influence skepticism toward AI, such as previous experiences, time, age, interests, and technology acceptance	[43,45,48,50-52]	High confidence	There were minor concerns regarding methodology and adequacy.
Workplace changes			
AI systems could save clinicians time through automation.	[43-48,50,53-55]	Moderate confidence	There were moderate concerns regarding coherence and minor concerns regarding methodology and adequacy, justifying one level of confidence downgrade.
Clinicians held differing perspectives on the importance of cost for AI system adoption	[47,48,54]	Moderate confidence	There were moderate concerns regarding adequacy and minor concerns regarding methodology, justifying one level of confidence downgrade.
There were conflicting views on the ideal level of AI system integration with existing clinical systems.	[45,47-50,52,55]	High confidence	There were minor concerns regarding methodology.
Technological concerns			
Clinician concerns regarding technological issues such as AI system or user errors.	[43-52,55]	High confidence	There were minor concerns regarding methodology.
Clinicians expressed a need for specific training in AI systems and being informed about AI technology	[47,48,52,55]	High confidence	There were minor concerns regarding methodology, coherence, and adequacy.
**Clinical impact**			
AI could provide valuable diagnostic support, increasing clinical effectiveness and accuracy.	[44,45,47-49,53-55]	High confidence	There were minor concerns regarding methodology and adequacy.
Desired features			
Clinicians expressed preferences for specific features in AI systems	[44-52,55]	High confidence	There were minor concerns regarding methodology and adequacy.
Clinicians emphasized the importance of AI systems being adaptable and customizable	[44,46,49,50]	High confidence	There were minor concerns regarding methodology.
User-friendliness of AI systems was emphasized by numerous clinicians as essential for adoption	[47-49,51,55]	High confidence	There were minor concerns regarding methodology and adequacy.
Bias			
Clinicians are concerned that AI could perpetuate biases from its training data or its creators.	[45,46,48,50-52]	High confidence	There were minor concerns regarding methodology and adequacy.
Clinicians fear over-relying on AI, leading to automation bias.	[45,46,48,51,52]	High confidence	There were minor concerns regarding methodology and adequacy.
Data security, privacy, and legal implications			
The security of patient data processed by AI is a significant concern for clinicians.	[43,45-48,50]	High confidence	There were minor concerns regarding methodology, coherence, and adequacy.
There are significant concerns regarding the legal liability and responsibility when using AI in clinical decisions.	[45,46,48,50,52,55]	High confidence	There were minor concerns regarding methodology and adequacy.

a CERQual: Confidence in Evidence from Reviews of Qualitative research.

b AI: artificial intelligence.

Details are provided in Multimedia Appendix 4.

## Discussion

### Principal Results

When synthesizing primary care clinician perspectives of various AI systems, 3 analytical themes emerged. The GRADE-CERQual assessment indicated high confidence in 15 findings, moderate confidence in 5 findings, and low confidence in one finding.

### The Human-Machine Relationship

There were many positive remarks on the potential for AI to assist clinicians in administrative tasks [45-47,51-53,55], clinical work [45,46,52,55], and education [51,53,54]. Some studies highlighted fears of AI replacing human roles [46,48,50]. Resistance to AI was noted, with concerns that AI might disrupt workflow and increase task time [43,47,48,50,53]. The underlying causes of skepticism were discussed. Some attributed it to previous negative experiences with other digital tools [51,52], others to their age or technical alignment [45,48,50]. Positive views on AI systems were generally seen in studies where AI tools were deemed effective, seamlessly integrated, and saved time [44,45,47].

### The Technologically Enhanced Clinic

Some clinicians saw benefits in automating tasks like documentation and consultation [44-48], whereas others feared potentially increased workload [43,47,50,53]. Technical concerns were found, including fears of computer errors or user errors [43-53,55]. Clinicians valued AI’s potential to assist in clinical tasks such as decision support [44,45,47-49,53-55]. Clinicians discussed desirable AI features, such as diagnostic support, integration with telemedicine, and customization options [44-52,55]. Several studies mentioned the importance of the system’s user-friendliness [47-49,51,55].

### The Societal Impact of AI

Security and privacy issues were highlighted, particularly regarding the handling of sensitive patient data and the risks of unauthorized access [43,45-48,50]. Societal impact, including potential biases and overreliance on AI, was concerns [45,46,48,50-52], and potential legal implications if clinicians acted outside of the AI recommendations [45,46,50,55]. Clinicians expressed greater confidence in adopting AI systems that had received formal regulatory approval or institutional endorsement [49,51].

### Comparison With Prior Work

Our findings regarding clinicians’ views of the potential of AI and reservations regarding safety aspects are similar to a previous systematic review of AI-powered chatbots for managing chronic illness, which provided insights into the usability and acceptance of AI in health care. The review found that participants gave positive feedback regarding perceived usefulness, satisfaction, and ease of use. The review also concluded that the safety of AI-powered chatbots has been overlooked and needs to be considered more thoroughly in future designs [56].

As AI expands into health care, a significant concern has emerged: the risk of bias. Since AI relies on historical data that could be statistically or socially biased, it could potentially incur a risk of worsening patient outcomes [57]. This coincides with our findings regarding clinicians’ concerns about biased AI systems.

In this review, clinicians were positive toward simplifying certain tasks using AI while simultaneously having concerns regarding technical aspects. Another systematic review of stakeholders’ perspectives on clinical AI implementation, which included perspectives of health care providers, similarly found that health care providers saw benefits in using AI for reducing repetitive tasks, improving patient outcomes, and clinical training. Reservations toward AI included implementation issues, uncertainty around its mechanics, and skepticism toward its ability to inform clinical decisions [58].

In the current review, opinions on whether clinicians should be involved in the development of AI systems were mixed. One scoping review published in 2020 found the engagement of clinicians in health care AI development and research to be crucial [59]. The ethical and regulatory challenges expressed in the findings of this review are also brought to light in a scoping review published in 2022. Their findings suggest that AI research and development in health care is currently outpacing the creation of supporting AI governance, and there is a need for international collaboration to facilitate comprehensive AI governance in this sector [60]. There were similar findings in another article published in 2021, where the author concludes that there is an apparent risk of regulations and oversight falling behind AI’s rapid development and integration [61].

This review focuses solely on clinicians, although many other professionals are implicated in the adoption of AI into health care. Further investigation of perspectives of information technology experts, managers, and other stakeholders could prove valuable in the development, adoption, and integration of AI systems [62].

### Strengths and Limitations

#### Strengths

To the best of the authors’ knowledge, no systematic review on this topic in a primary health care setting is currently available. The review provides new and valuable information on the topic. The review adhered to the PRISMA and ENTREQ guidelines, was pre-registered with PROSPERO, and searched across several large databases. Thematic synthesis was employed by two independent authors, enhancing reliability, validity, and reducing bias.

#### Limitations

The field of AI is rapidly expanding, and perspectives on AI in primary health care could swiftly change over time. Several new studies have emerged since this analysis was conducted; thus, further research is needed to better understand clinician perspectives on the latest AI advancements. This review relied on the authors’ definitions of AI in the included articles, potentially increasing the variability of the results. The limited geographic range of the included studies may affect the generalizability of the findings. In this review, we only included studies from high-income countries. Research on AI in health care in low- and middle-income countries is very limited, as most AI health systems are developed and tested in high-income countries. Additionally, no PRESS checklist for peer review of search strings was completed.

### Practical Implications and Identified Gaps in the Qualitative Literature

The findings of this review suggest that AI systems should focus on reducing administrative burden and supporting certain clinical tasks, provided they do not disrupt clinicians’ workflows. These systems must demonstrate time-saving capabilities and seamlessly integrate with existing infrastructure, such as electronic medical records. Through these functions, an AI system could enhance a medical visit by allowing the clinician to focus on patient contact rather than administrative tasks.

Continuous monitoring for computer errors, structured AI training programs for clinicians, and simplifying user interfaces are essential to minimize user errors. Additionally, ensuring robust data handling practices is critical to maintaining patient privacy and security. There is also a desire among clinicians for clearly defined medicolegal responsibilities.

Developers of health care-related AI systems should aim to mitigate system bias and consider collaborating with clinicians in the development process to build initial trust and address potential concerns. Involving clinicians with previous experiences of AI or CDSS in the development or integration of AI systems might facilitate adoption and use. Moreover, current AI tools should complement, not replace, clinical decision-making. It is important to provide younger clinicians with opportunities to develop critical reasoning skills without fostering over-reliance on AI-generated outputs.

The review revealed gaps regarding clinician perspectives on AI in primary health care, specifically in LLMs. Perspectives on ethical implications focused mainly on bias in AI systems, patient privacy and data security, medicolegal implications, transparency and accountability, and equity in AI system access. However, deeper analyses regarding the ethical implications of modern AI systems, including how AI might alter clinicians’ professional roles, authority, and autonomy, were scarce. Some of the included studies mentioned clinicians expressing thoughts on their reliance on AI systems and how their autonomy might be affected. However, further research could provide valuable information on these aspects that directly influence clinicians’ acceptance and utilization of AI technologies.

Findings from this review suggest that some clinicians are aware of long-term job implications and possible job displacement due to the introduction of AI into health care. Threats to the professional autonomy of clinicians could be due to automation bias, potentially overriding or deskilling clinical judgment with decision-support recommendations becoming default options, or by reducing the professional freedom of clinicians when many clinical actions are turned into data, enabling scrutiny of even the smallest decisions. Further research on AI’s effects on the evolution of clinicians’ career paths and future autonomy is warranted.

While many clinicians assume ultimate responsibility in patient care, several fear “legal whiplash” if they disregard an AI recommendation that later proved correct or followed one that proved harmful. These findings highlight the need for clear governance frameworks by having AI tools accredited and liability boundaries specified by a professional body such as the EU AI Act [63] and FDA Software-as-a-Medical-Device (SaMD) guidance [64]. Further empirical research is recommended to evaluate how such regulations translate into everyday primary care. Another possible pre-condition for ethically acceptable AI use could be provided by transparent AI reasoning paths, enabling clinicians to follow a defensible audit trail rather than a simple, final output that could prevent shared decision-making with patients.

### Future Directions

Further research on AI in primary health care is needed, especially in low- and middle-income countries. Notable gaps in the literature include evaluations of LLMs in primary health care, which are expected to have great potential. Ethnographic studies could yield deeper insights into AI’s impact on the professional role of clinicians and long-term career implications. Further in-depth, qualitative research on these topics could prove helpful for future AI system development and integration.

Future research should also expand beyond cross-sectional studies to longitudinal, mixed-methods studies that follow AI systems from adoption to routine use in primary care clinics for further in-depth analysis of AI use and a deeper understanding of facilitators and barriers to adoption. Additionally, systematic reviews targeting specific types of AI or clinical use cases would support a more nuanced understanding of AI implementation in diverse primary care contexts.

### Conclusions

Clinicians view AI as a technology that could both enhance and complicate primary health care. While AI can provide substantial support, its integration into health care requires careful consideration of ethical implications, technical reliability, and the maintenance of human oversight. Interpretation is constrained by heterogeneity in qualitative methods and the diverse AI technologies examined across studies. More in-depth qualitative research on the effects of AI on clinicians’ careers and autonomy could prove helpful for the future development of AI systems.

## Supplementary material

10.2196/72210 Multimedia Appendix 1 Search strategy.

10.2196/72210 Multimedia Appendix 2 Codebook and themes.

10.2196/72210 Multimedia Appendix 3 Critical Appraisal Skills Programme (CASP) checklist for Qualitative Research.

10.2196/72210 Multimedia Appendix 4 Evidence profile table of the Grading of Recommendations Assessment, Development, and Evaluation (GRADE)-Confidence in the Evidence from Reviews of Qualitative Research (CERQual) assessments.

10.2196/72210 Checklist 1 Enhancing transparency in reporting the synthesis of qualitative research (ENTREQ) checklist.

10.2196/72210 Checklist 2 Preferred Reporting Items for Systematic Reviews and Meta-Analyses (PRISMA) checklist.

10.2196/72210 Checklist 3 PRISMA-S checklist.

## Abbreviations

AI: artificial intelligence
CASP: critical appraisal skills program
CDSS: clinical decision support system
ENTREQ: Enhanced Transparency in Reporting the Synthesis of Qualitative Research
GAI: generative artificial intelligence
GP: general practitioner
GRADE-CERQual: Grading of Recommendations Assessment, Development, and Evaluation–Confidence in the Evidence From Reviews of Qualitative Research
LLM: large language model
ML: machine learning
NLP: natural language processing
PRISMA: Preferred Reporting Items for Systematic Reviews and Meta-Analyses
PRISMA-S: Preferred Reporting Items for Systematic Reviews and Meta-Analyses Search
SPIDER: Sample, Phenomenon of Interest, Design, Evaluation, and Research
