# Supplementary material for: Exploring Clinician Perspectives on Artificial Intelligence in Primary Care: Qualitative Systematic Review and Meta-Synthesis
Source: JMIR AI. 2026 Feb 5;5:e72210. doi: 10.2196/72210 (PMC12875425; doi:10.2196/72210)
Supplement: Multimedia Appendix 1 [file ai-v5-e72210-s001.doc]

Search string design and search strings for each database

Search string SPIDER design

| **SPIDER element** | **Operational definition** | **Controlled vocabulary terms** | **Free‑text terms** |
| --- | --- | --- | --- |
| **S – Sample** | Clinicians in primary‑care settings | MEDLINE (MeSH): “Primary Health Care”; “General Practice” CINAHL Headings: “Primary Health Care+”; “Family Practice+”; “General Practice+” | “primary care*”; “primary health care”; “family practi*”; “family practice”; “family medicine”; “general practi*” |
| **PI – Phenomenon of Interest** | Artificial‑intelligence tools used in primary care | MeSH: “Artificial Intelligence”; “Machine Learning”; “Natural Language Processing” CINAHL Headings: “Artificial Intelligence+”; “Machine Learning+”; “Natural Language Processing+” | “artificial intelligence*”; “AI”; “chatGPT”; “GPT”; “deep learning”; “neural network*”; “machine learning*”; “natural language processing*”; “large language model*”; “generative AI” |
| **D – Design** | Qualitative or mixed‑methods study designs | (Not restricted at search stage; qualitative eligibility applied during screening) | - |
| **E – Evaluation** | Clinician experiences, perspectives, attitudes | - | experienc*; perception*; impression*; opinion*; attitud*; reaction*; perspectiv*; satisfactio*; sentimen*; standpoin*; reflectio*; conceptio*; expectatio*; view* |
| **R – Research type** | Qualitative or mixed‑methods research | (Handled during screening) | - |

Dates: published from inception to February 5, 2024

Filters: SCOPUS was filtered for Publication stage: “Final”.

Search strings

MEDLINE (PubMed):

("Primary Health Care"[Mesh] OR "General Practice"[Mesh] OR "family practi*" OR "family medicine") AND ("Artificial Intelligence"[Mesh] OR "chatGPT" OR "GPT" OR "deep learning" OR "neural network" OR "machine learning" OR "natural language processing" OR "large language model*" OR "generative AI") AND (experienc* OR perception* OR impression* OR opinion* OR attitud* OR reaction* OR perspectiv* OR satisfactio* OR sentimen* OR standpoin* OR reflectio* OR conceptio* OR expectatio* OR view*)

Results: 394

SCOPUS:

TITLE-ABS-KEY ("primary health care" OR "primary care*" OR "general practi*" OR "family practi*" OR "family medicine") AND TITLE-ABS-KEY ("artificial intelligence*" OR "AI" OR "chatGPT" OR "GPT" OR "deep learning" OR "neural network" OR "machine learning*" OR "natural language processing*" OR "large language model*" OR "generative AI") AND TITLE-ABS-KEY (experienc* OR perception* OR impression* OR opinion* OR attitud* OR reaction* OR perspectiv* OR satisfactio* OR sentimen* OR standpoin* OR reflectio* OR conceptio* OR expectatio* OR view*)

Results: 665

Web of Science:

TS=(("primary health care" OR "primary healthcare" OR "primary care" OR

"family practice" OR "general practice" OR "family practi*" OR "family medicine")

AND

("artificial intelligence" OR "chatgpt" OR "gpt" OR "deep learning" OR

"neural network*" OR "machine learning" OR "natural language processing" OR

"large language model*" OR "generative ai")

AND

(experienc* OR perception* OR impression* OR opinion* OR attitud* OR reaction* OR

perspectiv* OR satisfactio* OR sentimen* OR standpoin* OR reflectio* OR

conceptio* OR expectatio* OR view*))

Results: 349

CINAHL (EBSCOhost):

( (MH "Primary Health Care+") OR (MH "Family Practice+") OR (MH "General Practice+") OR TI ("family practi*" OR "family medicine" OR "primary care") OR AB ("family practi*" OR "family medicine" OR "primary care") ) AND ( (MH "Artificial Intelligence+") OR (MH "Machine Learning+") OR (MH "Natural Language Processing+") OR TI ("chatGPT" OR "GPT" OR "deep learning" OR "neural network*" OR "machine learning" OR "natural language processing" OR "large language model*" OR "generative AI") OR AB ("chatGPT" OR "GPT" OR "deep learning" OR "neural network*" OR "machine learning" OR "natural language processing" OR "large language model*" OR "generative AI") ) AND ( TI (experienc* OR perception* OR impression* OR opinion* OR attitud* OR reaction* OR perspectiv* OR satisfactio* OR sentimen* OR standpoin* OR reflectio* OR conceptio* OR expectatio*) OR AB (experienc* OR perception* OR impression* OR opinion* OR attitud* OR reaction* OR perspectiv* OR satisfactio* OR sentimen* OR standpoin* OR reflectio* OR conceptio* OR expectatio* OR view*) )

Results: 84
