# Supplementary material for: Exploring Clinician Perspectives on Artificial Intelligence in Primary Care: Qualitative Systematic Review and Meta-Synthesis
Source: JMIR AI. 2026 Feb 5;5:e72210. doi: 10.2196/72210 (PMC12875425; doi:10.2196/72210)
Supplement: Multimedia Appendix 2 [file ai-v5-e72210-s002.docx]

Codebook

| **Name** | **Descriptive theme alignment** | **Studies** | **References** |
| --- | --- | --- | --- |
| Administrative support desired |  | 1 | 1 |
| AI accuracy concerns |  | 1 | 2 |
| AI adaptation to clinicians |  | 1 | 2 |
| AI advantages over older technology |  | 1 | 2 |
| AI as a virtual assistant |  | 1 | 2 |
| AI assisted empathy |  | 2 | 2 |
| AI difficulties managing complex issues |  | 1 | 1 |
| AI disrupting traditional consultation models |  | 1 | 1 |
| AI easier to trust if the system is scientifically proven to work |  | 1 | 1 |
| AI enabling clinicians to focus on clinical tasks |  | 5 | 11 |
| AI error concerns |  | 1 | 1 |
| AI especially important in community health centers |  | 1 | 1 |
| AI facilitating decision of level of care |  | 1 | 5 |
| AI flagging important information |  | 1 | 1 |
| AI having a broad knowledge base |  | 1 | 1 |
| AI helpful in keeping track of guidelines |  | 1 | 1 |
| AI helpful in very large populations |  | 1 | 1 |
| AI improving patient follow up |  | 2 | 3 |
| AI improving patients trust in the clinician |  | 1 | 1 |
| AI in preventative care needs appropriate integration |  | 1 | 2 |
| AI increasing patient confidence in clinician |  | 2 | 2 |
| AI lack of empathy |  | 2 | 8 |
| AI lack of human competencies |  | 1 | 1 |
| AI leading to earlier detection and treatment |  | 1 | 1 |
| AI optimizing care through administrative assistance |  | 1 | 2 |
| AI processing speed |  | 1 | 1 |
| AI promoting universialisation of diagnostics |  | 1 | 1 |
| AI providing a second opinion |  | 3 | 11 |
| AI replacing clinicians |  | 1 | 1 |
| AI risk for patient safety |  | 1 | 2 |
| AI role in improving health |  | 1 | 1 |
| AI should be able to predict surges in visits |  | 1 | 1 |
| AI should enhance and not replace patient-provider relationship |  | 1 | 1 |
| AI system providing quick results |  | 1 | 1 |
| AI to optimize scheduling |  | 1 | 1 |
| AI triage could limit access to patients unable to use technology |  | 2 | 3 |
| AI use increasing diagnostic accuracy |  | 2 | 2 |
| AI use leading to increased clinician confidence |  | 1 | 1 |
| Algorithm use equated to screening use |  | 1 | 1 |
| Algorithmic bias |  | 1 | 1 |
| Annoying or unhelpful prompts |  | 1 | 1 |
| Automate referrals |  | 5 | 11 |
| Automation bias |  | 1 | 1 |
| Awareness of the limitations of AI |  | 1 | 1 |
| Bad experience with EHR causing skepticism towards AI systems |  | 2 | 4 |
| Benefits of automation |  | 1 | 1 |
| Benefits of remote usage |  | 1 | 1 |
| Careful with AI as to not lose advantages of older models of care |  | 1 | 4 |
| Causing an increased workload |  | 1 | 1 |
| CDSS performance is user driven |  | 2 | 12 |
| Changing physician-patient relationship |  | 6 | 12 |
| Clinical decision support |  | 1 | 1 |
| Clinicans rely more on gut feeling than AI system |  | 1 | 2 |
| Clinician age group differences in attitude towards AI |  | 4 | 6 |
| Clinician concerns regarding technology training |  | 2 | 6 |
| Clinician control over AI usage |  | 1 | 4 |
| Clinician control over medical records |  | 1 | 1 |
| Clinician insight in AI system |  | 1 | 1 |
| Clinician lack of knowledge about AI causing lack of ideas for features |  | 1 | 1 |
| Clinician-AI collaboration oppurtunities rather than distrust |  | 1 | 3 |
| Computer problems hindering AI usage |  | 2 | 8 |
| Computer training importance |  | 1 | 3 |
| Concerns regarding AI differentiating relevance of data |  | 2 | 3 |
| Concerns regarding technical issues |  | 1 | 3 |
| Consultation preparation |  | 2 | 2 |
| Control over implementation |  | 2 | 4 |
| Cost saving |  | 1 | 1 |
| Customizable depth of information |  | 4 | 14 |
| Data security and privacy concerns |  | 1 | 2 |
| Demographic bias |  | 1 | 1 |
| Desensitisation to support system risks increases in clinician error |  | 1 | 1 |
| Desire for AI to enhance team-based care |  | 1 | 1 |
| Desire for system augmentation to increase performance |  | 1 | 1 |
| Diagnostic efficiency benefit |  | 1 | 1 |
| Difference in information interpretation between clinician and AI |  | 2 | 4 |
| Disrupting workflow |  | 1 | 3 |
| Doctor exposure and safety concerns |  | 1 | 1 |
| Explainability of an AI |  | 1 | 1 |
| Extracting specific information |  | 2 | 4 |
| Facilitating doctor-patient communication |  | 1 | 10 |
| Facilitating documentation |  | 1 | 1 |
| Fear of not knowing how to use the system in front of patients |  | 3 | 3 |
| Fear of user error |  | 1 | 1 |
| Few reservations towards AI |  | 3 | 3 |
| Following science and guidelines |  | 1 | 1 |
| Gathering and relaying information from algorithm could be taxing |  | 1 | 1 |
| Growing use of similar algorithms may facilitate implementation of algorithm |  | 1 | 1 |
| Have not found a useful DSS |  | 1 | 1 |
| Help with information gathering |  | 1 | 2 |
| Helpful in professional development |  | 1 | 1 |
| Identify patients with high risk of disease |  | 1 | 1 |
| Importance of accessibility |  | 1 | 1 |
| Importance of avoiding redundancies between algorithm and existing tools |  | 1 | 2 |
| Importance of clear communication about algorithm results to avoid confusing or alarming patients |  | 5 | 17 |
| Importance of ease of use |  | 1 | 1 |
| Importance of system content |  | 2 | 3 |
| Importance of system cost |  | 2 | 5 |
| Improving clinical consultation |  | 3 | 4 |
| Improving patient care |  | 1 | 2 |
| Increased diagnostic accuracy with AI |  | 1 | 1 |
| Institutional influence over AI usage |  | 1 | 1 |
| Interest in applying AI to population-level datasets |  | 1 | 1 |
| Interest in remote monitoring and self-management AI systems |  | 1 | 2 |
| Introducing AI to already trained clinicians so to not loose traditional training |  | 1 | 1 |
| Involvement in technology development |  | 1 | 7 |
| Journal summarization assistance |  | 2 | 3 |
| Lack of a certain feature in DSS |  | 1 | 2 |
| Lack of social connection to AI |  | 4 | 7 |
| Legal concerns |  | 1 | 1 |
| Limited time and resources are barriers to system implementation |  | 2 | 2 |
| Little change in workflow |  | 1 | 1 |
| Machine learning bias |  | 1 | 1 |
| Malicious use by pharmaceutical companies |  | 1 | 1 |
| Moving away from a protectionist model |  | 1 | 1 |
| Need for AI to be more accurate than humans to not be obsolete |  | 1 | 1 |
| Need for AI to reduce workload |  | 1 | 1 |
| Need for clinician complement to AI system |  | 3 | 8 |
| Need for customizability and adaptability |  | 1 | 1 |
| Need for predetermined policies and routines before adoption |  | 1 | 1 |
| Need for prescription support |  | 2 | 2 |
| Need to clarify medicolegal liability |  | 1 | 1 |
| Negative effects of replacing the act of writing a medical journal |  | 1 | 1 |
| Negative social effects |  | 1 | 1 |
| No need for detailed data from algorithm |  | 2 | 3 |
| No threat of replacing doctors |  | 1 | 1 |
| Non intuitive usage |  | 1 | 2 |
| Not involved in the system |  | 1 | 2 |
| Ok with AI functioning without knowing how |  | 1 | 1 |
| Participation only on a voluntary basis |  | 1 | 1 |
| Patient approval of AI is expected |  | 1 | 1 |
| Patient care augmented by new technologies |  | 2 | 2 |
| Patient triage as a possible feature |  | 1 | 1 |
| Patients expect AI technologies |  | 1 | 1 |
| Personalized AI training |  | 1 | 2 |
| Physician satisfaction not taken into consideration when developing technology |  | 2 | 2 |
| Physician technical inclination affecting AI usage |  | 1 | 1 |
| Positive disease progression influence |  | 1 | 1 |
| Positive non-human traits |  | 1 | 1 |
| Positivity towards portability of technology |  | 6 | 22 |
| Potential for saving time |  | 1 | 1 |
| Potential to be useful in relation to medical records |  | 1 | 1 |
| Prefering patient care focus rather than economic gain with the use of technology |  | 1 | 1 |
| Priority of AI systems should be documentation, practice operations and triage |  | 1 | 1 |
| Privacy issues negatively impact provider-patient relationship |  | 1 | 2 |
| Progressive implementation leads to higher acceptance |  | 1 | 1 |
| Provide guidance in real time during consult |  | 1 | 1 |
| Providers concern for patient privacy |  | 1 | 1 |
| Providers thoughts on how to present data to patients |  | 1 | 1 |
| Providers wish to maintain contact with patient during presentation of algorithm results |  | 3 | 4 |
| Providing guidance and learning |  | 1 | 1 |
| Recall patients for testing |  | 1 | 1 |
| Recommended guidelines are not applicable for all patients |  | 2 | 2 |
| Reduced workload |  | 3 | 7 |
| Reducing administrative burden |  | 2 | 4 |
| Resistance to technological change |  | 1 | 5 |
| Social and collegial influence on CDSS use |  | 1 | 1 |
| Some populations might not have access to technology needed for algorithm use |  | 6 | 13 |
| System integration concerns |  | 1 | 1 |
| System separation |  | 1 | 1 |
| System usage fatigue |  | 1 | 3 |
| Technological development is necessary |  | 1 | 1 |
| Technology enables work outside of the consultation |  | 2 | 3 |
| Telehealth combined with AI is useful |  | 1 | 1 |
| Too broad recommendations |  | 3 | 13 |
| Trust in AI |  | 1 | 1 |
| Trust in AI related to validation from other healthcare professionals |  | 1 | 1 |
| Trust in creator of AI |  | 1 | 1 |
| Unable to continue if the system goes down |  | 1 | 3 |
| Uncomfortable to use in front of patients |  | 4 | 9 |
| Use of AI causing additional time expenditure |  | 1 | 1 |
| Use of AI causing increased patient expectations |  | 1 | 2 |
| Use of AI leading to monitorization of physicians |  | 1 | 1 |
| Wish for dedicated time for AI usage |  | 1 | 1 |

Descriptive themes

| Technological concerns | | | Data security, privacy and legal implications | | | Interaction with AI | | | Bias | | |  |
| --- | --- | --- | --- | --- | --- | --- | --- | --- | --- | --- | --- | --- |
|  |  |  |  |  |  |  |  |  |  |  |  |  |
|  |  |  |  |  |  |  |  |  |  |  |  |  |
| Clinical impact | | | Resistance to AI | | | Workplace changes | | | Desired AI features | | |  |
|  |  |  |  |  |  |  |  |  |  |  |  |  |

Analytical themes

| IA | RA |  |  |
| --- | --- | --- | --- |
| The human-machine relationship | | |  |
|  |  |  |  |

| CI | DAF |  |  |
| --- | --- | --- | --- |
| TC | WC |  |  |
| The technologically enhanced clinic | | |  |
|  |  |  |  |

| B | DPLI |  |  |
| --- | --- | --- | --- |
| The societal impact of AI | | |  |
|  |  |  |  |
