# Supplementary material for: Exploring Clinician Perspectives on Artificial Intelligence in Primary Care: Qualitative Systematic Review and Meta-Synthesis
Source: JMIR AI. 2026 Feb 5;5:e72210. doi: 10.2196/72210 (PMC12875425; doi:10.2196/72210)
Supplement: Multimedia Appendix 3 [file ai-v5-e72210-s003.doc]

CASP Critical Appraisal Checklist for Qualitative Research

|  | Q1 | Q2 | Q3 | Q4 | Q5 | Q6 | Q7 | Q8 | Q9 | Q10 | Sum of Y |
| --- | --- | --- | --- | --- | --- | --- | --- | --- | --- | --- | --- |
| Davis M et al., 2024 [43] | Y | Y | Y | Y | Y | N | Y | Y | Y | Y | 9 |
| Litvin CB et al., 2012 [44] | Y | Y | Y | N | Y | N | CT | CT | Y | Y | 6 |
| Navarro DF et al., 2023 [45] | Y | Y | Y | Y | Y | CT | Y | Y | Y | Y | 9 |
| Kocaballi AB et al., 2020 [46] | Y | Y | Y | Y | Y | CT | Y | Y | Y | Y | 9 |
| Shibl R et al., 2013 [47] | Y | Y | Y | Y | Y | CT | Y | Y | Y | Y | 9 |
| Buck C et al., 2022 [48] | Y | Y | Y | Y | Y | CT | CT | Y | Y | Y | 8 |
| Ahearn MD et al., 2003 [49] | Y | Y | Y | Y | Y | CT | CT | Y | Y | Y | 8 |
| Allen MR et al., 2024 [50] | Y | Y | Y | Y | Y | CT | Y | Y | Y | Y | 9 |
| Nash D et al., 2023 [51] | Y | Y | Y | Y | Y | Y | CT | Y | Y | Y | 9 |
| Upshaw T et al., 2023 [52] | Y | Y | Y | Y | Y | N | Y | Y | Y | Y | 9 |
| Libon J et al., 2023 [53] | Y | Y | Y | Y | Y | N | Y | Y | Y | Y | 9 |
| Sangers T et al., 2023 [54] | Y | Y | Y | Y | Y | CT | Y | Y | Y | Y | 9 |
| Helenason J et al., 2023 [55] | Y | Y | Y | Y | Y | N | Y | Y | Y | Y | 9 |

Y: Yes; N: NO; CT: Cannot tell.

Q1 Was there a clear statement of the aims of the research?

Q2 Is a qualitative methodology appropriate?

Q3 Was the research design appropriate to address the aims of the research?

Q4 Was the recruitment strategy appropriate to the aims of the research?

Q5 Was the data collected in a way that addressed the research issue?

Q6 Has the relationship between researcher and participants been adequately considered?

Q7 Have ethical issues been taken into consideration?

Q8 Was the data analysis sufficiently rigorous?

Q9 Is there a clear statement of findings?

Q10 How valuable is the research?

References

43. Davis M, Dysart GC, Doupnik SK, Hamm ME, Schwartz KTG, George-Milford B, et al. Adolescent, Parent, and Provider Perceptions of a Predictive Algorithm to Identify Adolescent Suicide Risk in Primary Care. Academic Pediatrics. 2024 2024/05/01/;24(4):645-53. doi: https://doi.org/10.1016/j.acap.2023.12.015.

44. Litvin CB, Ornstein SM, Wessell AM, Nemeth LS, Nietert PJ. Adoption of a clinical decision support system to promote judicious use of antibiotics for acute respiratory infections in primary care. International Journal of Medical Informatics. 2012 2012/08/01/;81(8):521-6. doi: https://doi.org/10.1016/j.ijmedinf.2012.03.002.

45. Navarro DF, Kocaballi AB, Dras M, Berkovsky S. Collaboration, not Confrontation: Understanding General Practitioners’ Attitudes Towards Natural Language and Text Automation in Clinical Practice. ACM Transactions on Computer-Human Interaction. 2023;30(2):Article 29. doi: 10.1145/3569893.

46. Kocaballi AB, Ijaz K, Laranjo L, Quiroz JC, Rezazadegan D, Tong HL, et al. Envisioning an artificial intelligence documentation assistant for future primary care consultations: A co-design study with general practitioners. J Am Med Inform Assoc. 2020 Nov 1;27(11):1695-704. PMID: 32845984. doi: 10.1093/jamia/ocaa131.

47. Shibl R, Lawley M, Debuse J. Factors influencing decision support system acceptance. Decision Support Systems. 2013 2013/01/01/;54(2):953-61. doi: https://doi.org/10.1016/j.dss.2012.09.018.

48. Buck C, Doctor E, Hennrich J, Jöhnk J, Eymann T. General Practitioners' Attitudes Toward Artificial Intelligence-Enabled Systems: Interview Study. J Med Internet Res. 2022 Jan 27;24(1):e28916. PMID: 35084342. doi: 10.2196/28916.

49. Ahearn MD, Kerr SJ. General practitioners' perceptions of the pharmaceutical decision-support tools in their prescribing software. Med J Aust. 2003 Jul 7;179(1):34-7. PMID: 12831382. doi: 10.5694/j.1326-5377.2003.tb05415.x.

50. Allen MR, Webb S, Mandvi A, Frieden M, Tai-Seale M, Kallenberg G. Navigating the doctor-patient-AI relationship - a mixed-methods study of physician attitudes toward artificial intelligence in primary care. BMC Primary Care. 2024 Jan 27;25(1):42. PMID: 38281026. doi: 10.1186/s12875-024-02282-y.

51. Nash DM, Thorpe C, Brown JB, Kueper JK, Rayner J, Lizotte DJ, et al. Perceptions of Artificial Intelligence Use in Primary Care: A Qualitative Study with Providers and Staff of Ontario Community Health Centres. The Journal of the American Board of Family Medicine. 2023;36(2):221-8. doi: 10.3122/jabfm.2022.220177R2.

52. Upshaw TL, Craig-Neil A, Macklin J, Gray CS, Chan TCY, Gibson J, et al. Priorities for Artificial Intelligence Applications in Primary Care: A Canadian Deliberative Dialogue with Patients, Providers, and Health System Leaders. The Journal of the American Board of Family Medicine. 2023;36(2):210-20. doi: 10.3122/jabfm.2022.220171R1.

53. Libon J, Ng C, Bailey A, Hareendranathan A, Joseph R, Dulai S. Remote diagnostic imaging using artificial intelligence for diagnosing hip dysplasia in infants: Results from a mixed-methods feasibility pilot study. Paediatrics & Child Health. 2023;28(5):285-90. doi: 10.1093/pch/pxad013.

54. Sangers TE, Wakkee M, Moolenburgh FJ, Nijsten T, Lugtenberg M. Towards successful implementation of artificial intelligence in skin cancer care: a qualitative study exploring the views of dermatologists and general practitioners. Archives of Dermatological Research. 2023 2023/07/01;315(5):1187-95. doi: 10.1007/s00403-022-02492-3.

55. Helenason J, Ekström C, Falk M, Papachristou P. Exploring the feasibility of an artificial intelligence based clinical decision support system for cutaneous melanoma detection in primary care – a mixed method study. Scandinavian Journal of Primary Health Care. 2024 2024/01/02;42(1):51-60. doi: 10.1080/02813432.2023.2283190.
