# Supplementary material for: Exploring Clinician Perspectives on Artificial Intelligence in Primary Care: Qualitative Systematic Review and Meta-Synthesis
Source: JMIR AI. 2026 Feb 5;5:e72210. doi: 10.2196/72210 (PMC12875425; doi:10.2196/72210)
Supplement: Multimedia Appendix 4 [file ai-v5-e72210-s004.docx]

GRADE CERQual Evidence Profile table

| **Summary of review finding** | **Ref^a^** | **Methodological limitations** | **Coherence** | **Adequacy** | **Relevance** | ***CERQual assessment of confidence in the evidence*** | ***Explanation of CERQual assessment*** |
| --- | --- | --- | --- | --- | --- | --- | --- |
| **Interaction with AI** | | | | | | | |
| 1. Clinicians perceive AI as an assistant that could alleviate the burden of specific tasks. | [45-47, 51-55] | No/Very minor concerns  **Explanation:** The finding is supported by studies with very minor methodological limitations. | No/Very minor concerns  **Explanation:** High consistency across studies, with no significant contradictions identified. | Minor concerns  **Explanation:** Substantial volume and good depth of qualitative data supporting the finding. Three studies had a moderate volume of data [51, 52, 54] and one study had a small amount of data [53]. | No/Very minor concerns  **Explanation:** Directly relevant. The finding reflects clinicians’ perspectives on AI without mismatch in population, phenomenon, or setting. | High confidence | There were minor concerns regarding adequacy. |
| 2. AI may negatively impact the clinician-patient relationship due to a lack of human connection. | [46, 48] | Minor concerns  **Explanation:** The finding is supported by one study with very minor methodological limitations and one with minor limitations [48]. | Moderate concerns  **Explanation:** Significant contradiction in finding #3. | Serious concerns  **Explanation:** Small volume but good depth of qualitative data, provided by two studies. | No/Very minor concerns  **Explanation:** Directly relevant. The finding reflects clinicians’ perspectives on AI without mismatch in population, phenomenon, or setting. | Low confidence | There were serious concerns regarding adequacy, moderate concerns regarding coherence, and minor concerns regarding methodology, justifying two levels of confidence downgrade. |
| 3. AI could enhance clinician empathy or facilitate clinician-patient communication and confidence. | [43, 45, 46, 50, 52-55] | No/Very minor concerns  **Explanation:** The finding is supported by studies with very minor methodological limitations. | Moderate concerns  **Explanation:** Significant contradiction in finding #2. | Minor concerns  **Explanation:** Substantial volume and good depth of qualitative data supporting the finding. Three studies had a moderate volume of data [43, 52, 54] and one study had a small amount of data [53]. | No/Very minor concerns  **Explanation:** Directly relevant. The finding reflects clinicians’ perspectives on AI without mismatch in population, phenomenon, or setting. | Moderate confidence | There were moderate concerns regarding coherence and minor concerns regarding adequacy, justifying one level of confidence downgrade. |
| 4. Clinicians wish to retain control over AI systems and understand how they function. | [43, 45, 46, 48, 50, 55] | Minor concerns  **Explanation:** The finding is supported primarily by studies with very minor methodological limitations and one study with minor limitations [48]. | Minor concerns  **Explanation:** High consistency across studies, with some contradictions regarding the need to understand AI systems. | Minor concerns  **Explanation:** Substantial volume and good depth of qualitative data supporting the finding. One study had a moderate volume of data [43]. | No/Very minor concerns  **Explanation:** Directly relevant. The finding reflects clinicians’ perspectives on AI without mismatch in population, phenomenon, or setting. | High confidence | There were moderate concerns regarding adequacy, and minor concerns regarding methodology and coherence. |
| 5. Clinicians expressed conflicting views regarding trust in AI. | [45, 47, 55] | No/Very minor concerns  **Explanation:** The finding is supported by studies with very minor methodological limitations | No/Very minor concerns  **Explanation:** High consistency across studies, with no significant contradictions identified. | Moderate concerns  **Explanation:** Good depth of qualitative data, although data were provided by only three studies. | No/Very minor concerns  **Explanation:** Directly relevant. The finding reflects clinicians’ perspectives on AI without mismatch in population, phenomenon, or setting. | High confidence | There were moderate concerns regarding adequacy. The concern was not deemed serious enough for a downgrade of confidence. |

| **Summary of review finding** | **Ref** | **Methodological limitations** | **Coherence** | **Adequacy** | **Relevance** | ***CERQual assessment of confidence in the evidence*** | ***Explanation of CERQual assessment*** |
| --- | --- | --- | --- | --- | --- | --- | --- |
| **Resistance to AI** | | | | | | | |
| 6. Some clinicians fear being replaced or having their role diminished by AI. | [46, 48, 50] | Minor concerns  **Explanation:** The finding is supported by two studies with very minor methodological limitations and one study with minor limitations [48]. | Minor concerns  **Explanation:** High consistency across studies, with no significant contradictions identified. | Moderate concerns  **Explanation:** Good depth of qualitative data, although data were provided by only three studies. | No/Very minor concerns  **Explanation:** Directly relevant. The finding reflects clinicians’ perspectives on AI without mismatch in population, phenomenon, or setting. | Moderate confidence | There were moderate concerns regarding adequacy, and minor concerns regarding methodology and coherence, justifying one level of confidence downgrade. |
| 7. The introduction of AI could increase clinicians' time expenditure or disrupt workflows. | [43, 44, 47, 48, 50] | Minor concerns  **Explanation:** The finding is primarily supported by studies with very minor methodological limitations. One study had moderate limitations (recruitment strategy, incomplete reflexivity, and ethics reporting) [44]. | Moderate concerns  **Explanation:** The finding is contradicted by finding #9. | Minor concerns  **Explanation:** Substantial volume and good depth of qualitative data supporting the finding. One study provided a moderate volume of data [43]. | No/Very minor concerns  **Explanation:** Directly relevant. The finding reflects clinicians’ perspectives on AI without mismatch in population, phenomenon, or setting. | Moderate confidence | There were moderate concerns regarding coherence and minor concerns regarding methodology and adequacy, justifying one level of confidence downgrade. |
| 8. Multiple factors influence skepticism towards AI, such as previous experiences, time, age, interests and technology acceptance | [43, 45, 48, 50-52] | Minor concerns  **Explanation:** The finding is primarily supported by studies with very minor methodological limitations. One study had minor limitations [48]. | No/Very minor concerns  **Explanation:** High consistency across studies, with no significant contradictions identified. | Minor concerns  **Explanation:** Substantial volume and good depth of qualitative data supporting the finding. Three studies provided a moderate volume of data [43, 51, 52]. | No/Very minor concerns  **Explanation:** Directly relevant. The finding reflects clinicians’ perspectives on AI without mismatch in population, phenomenon, or setting. | High confidence | There were minor concerns regarding methodology and adequacy. |

| **Summary of review finding** | **Ref** | **Methodological limitations** | **Coherence** | **Adequacy** | **Relevance** | ***CERQual assessment of confidence in the evidence*** | ***Explanation of CERQual assessment*** |
| --- | --- | --- | --- | --- | --- | --- | --- |
| **Workplace changes** | | | | | | | |
| 9. AI systems could save clinicians time through automation. | [43-48, 50, 53-55] | Minor concerns  **Explanation:** The finding is supported primarily studies with very minor methodological limitations. One study had moderate limitations (recruitment strategy, incomplete reflexivity, and ethics reporting) [44]. Another study had minor limitations [48]. | Moderate concerns  **Explanation:** The finding is contradicted by finding #7. | Minor concerns  **Explanation:** Substantial volume and good depth of qualitative data supporting the finding. Two studies provided a moderate volume of data [43, 54] and one study provided a small volume of data [53]. | No/Very minor concerns  **Explanation:** Directly relevant. The finding reflects clinicians’ perspectives on AI without mismatch in population, phenomenon, or setting. | Moderate confidence | There were moderate concerns regarding coherence and minor concerns regarding methodology and adequacy, justifying one level of confidence downgrade. |
| 10. Clinicians held differing perspectives on the importance of cost for AI system adoption | [47, 48, 54] | Minor concerns  Explanation: The finding is supported by two studies with very minor methodological limitations and one study with minor limitations [48]. | No/Very minor concerns  **Explanation:** High consistency and without significant contradictions throughout the included studies. | Moderate concerns  **Explanation:** Good depth of qualitative data, although data is provided from only three studies. One study provided a moderate volume of data [54]. | No/Very minor concerns  **Explanation:** Directly relevant. The finding reflects clinicians’ perspectives on AI without mismatch in population, phenomenon, or setting. | Moderate confidence | There were moderate concerns regarding adequacy and minor concerns regarding methodology, justifying one level of confidence downgrade. |
| 11. There were conflicting views on the ideal level of AI system integration with existing clinical systems. | [45, 47-50, 52, 55] | Minor concerns  **Explanation:** The finding is primarily supported by studies with very minor methodological limitations. Two studies had minor limitations [48, 49]. | No/Very minor concerns  **Explanation:** High consistency and without significant contradictions throughout the included studies. | No/Very minor concerns  **Explanation:** Substantial volume and good depth of qualitative data supporting the finding. One study provided a moderate volume of data [52]. | No/Very minor concerns  **Explanation:** Directly relevant. The finding reflects clinicians’ perspectives on AI without mismatch in population, phenomenon, or setting. | High confidence | There were minor concerns regarding methodology. |

| **Summary of review finding** | **Ref** | **Methodological limitations** | **Coherence** | **Adequacy** | **Relevance** | ***CERQual assessment of confidence in the evidence*** | ***Explanation of CERQual assessment*** |
| --- | --- | --- | --- | --- | --- | --- | --- |
| **Technological concerns** | | | | | | | |
| 12. Clinician concerns regarding technological issues such as AI system or user errors. | [43-52, 55] | Minor concerns  **Explanation:** The finding is supported primarily studies with very minor methodological limitations. One study had moderate limitations (recruitment strategy, incomplete reflexivity, and ethics reporting) [44] and two studies had minor limitations [48,49]. | No/Very minor concerns  **Explanation:** High consistency and without significant contradictions throughout the included studies. | No/Very minor concerns  **Explanation:** Substantial volume and good depth of qualitative data supporting the finding. Three studies provided a moderate volume of data [43, 51, 52]. | No/Very minor concerns  **Explanation:** Directly relevant. The finding reflects clinicians’ perspectives on AI without mismatch in population, phenomenon, or setting. | High confidence | There were minor concerns regarding methodology. |
| 13. Clinicians expressed a need for specific training in AI systems and being informed about AI technology | [47, 48, 52, 55] | Minor concerns  **Explanation:** The finding is primarily supported by studies with very minor methodological limitations. One study had minor limitations [48]. | Minor concerns  **Explanation:** High consistency with only one significant contradiction throughout the included studies. | Minor concerns  **Explanation:** Substantial volume and good depth of qualitative data supporting the finding. One study provided a moderate volume of data [52]. | No/Very minor concerns  **Explanation:** Directly relevant. The finding reflects clinicians’ perspectives on AI without mismatch in population, phenomenon, or setting. | High confidence | There were minor concerns regarding methodology, coherence and adequacy. |

| **Summary of review finding** | **Ref** | **Methodological limitations** | **Coherence** | **Adequacy** | **Relevance** | ***CERQual assessment of confidence in the evidence*** | ***Explanation of CERQual assessment*** |
| --- | --- | --- | --- | --- | --- | --- | --- |
| **Clinical impact** | | | | | | | |
| 14. AI could provide valuable diagnostic support, increasing clinical effectiveness and accuracy. | [44, 45, 47-49, 53-55] | Minor concerns  **Explanation:** The finding is primarily supported by studies with very minor methodological limitations. One study had moderate limitations (recruitment strategy, incomplete reflexivity, and ethics reporting) [44] and two studies had minor limitations [48, 49]. | No/Very minor concerns  **Explanation:** High consistency and without significant contradictions throughout the included studies. | Minor concerns  **Explanation:** Substantial volume and good depth of qualitative data supporting the finding. One study provided a small volume of data [53]. | No/Very minor concerns  **Explanation:** Directly relevant. The finding reflects clinicians’ perspectives on AI without mismatch in population, phenomenon, or setting. | High confidence | There were minor concerns regarding methodology and adequacy. |

| **Summary of review finding** | **Ref** | **Methodological limitations** | **Coherence** | **Adequacy** | **Relevance** | ***CERQual assessment of confidence in the evidence*** | ***Explanation of CERQual assessment*** |
| --- | --- | --- | --- | --- | --- | --- | --- |
| **Desired features** | | | | | | | |
| 15. Clinicians expressed preferences for specific features in AI systems | [44-52, 55] | Minor concerns  **Explanation:** The finding is primarily supported by studies with very minor methodological limitations. One study had moderate limitations (recruitment strategy, incomplete reflexivity, and ethics reporting) [44] and two studies had minor limitations [48, 49]. | No/Very minor concerns  **Explanation:** High consistency and without significant contradictions throughout the included studies. | Minor concerns  **Explanation:** Substantial volume and good depth of qualitative data supporting the finding. Two studies provided a moderate volume of data [51, 52]. | No/Very minor concerns  **Explanation:** Directly relevant. The finding reflects clinicians’ perspectives on AI without mismatch in population, phenomenon, or setting. | High confidence | There were minor concerns regarding methodology and adequacy. |
| 16. Clinicians emphasized the importance of AI systems being adaptable and customizable | [44, 46, 49, 50] | Minor concerns  **Explanation:** The finding is primarily supported by studies with very minor methodological limitations. One study had moderate limitations (recruitment strategy, incomplete reflexivity, and ethics reporting) [44] and one study had minor limitations [49]. | No/Very minor concerns  **Explanation:** High consistency and without significant contradictions throughout the included studies. | No/Very minor concerns  **Explanation:** Substantial volume and good depth of qualitative data supporting the finding. | No/Very minor concerns  **Explanation:** Directly relevant. The finding reflects clinicians’ perspectives on AI without mismatch in population, phenomenon, or setting. | High confidence | There were minor concerns regarding methodology. |
| 17. User-friendliness of AI systems was emphasized by numerous clinicians as essential for adoption | [47-49, 51, 55] | Minor concerns  **Explanation:** The finding is primarily supported by studies with very minor methodological limitations. Two studies had minor limitations [48, 49]. | No/Very minor concerns  **Explanation:** High consistency and without significant contradictions throughout the included studies. | Minor concerns  **Explanation:** Substantial volume and good depth of qualitative data supporting the finding. One study provided a moderate volume of data [51]. | No/Very minor concerns  **Explanation:** Directly relevant. The finding reflects clinicians’ perspectives on AI without mismatch in population, phenomenon, or setting. | High confidence | There were minor concerns regarding methodology and adequacy. |

| **Summary of review finding** | **Ref** | **Methodological limitations** | **Coherence** | **Adequacy** | **Relevance** | ***CERQual assessment of confidence in the evidence*** | ***Explanation of CERQual assessment*** |
| --- | --- | --- | --- | --- | --- | --- | --- |
| **Bias** | | | | | | | |
| 18. Clinicians are concerned that AI could perpetuate biases from its training data or its creators. | [45, 46, 48, 50-52] | Minor concerns  **Explanation:** The finding is primarily supported by studies with very minor methodological limitations. One study had minor limitations [48]. | No/Very minor concerns  **Explanation:** High consistency and without significant contradictions throughout the included studies. | Minor concerns  **Explanation:** Substantial volume and good depth of qualitative data supporting the finding. Two studies provided a moderate volume of data [51, 52]. | No/Very minor concerns  **Explanation:** Directly relevant. The finding reflects clinicians’ perspectives on AI without mismatch in population, phenomenon, or setting. | High confidence | There were minor concerns regarding methodology and adequacy. |
| 19. Clinicians fear over-relying on AI, leading to automation bias. | [45, 46, 48, 51, 52] | Minor concerns  **Explanation:** The finding is primarily supported by studies with very minor methodological limitations. One study had minor limitations [48]. | No/Very minor concerns  **Explanation:** High consistency and without significant contradictions throughout the included studies. | Minor concerns  **Explanation:** Substantial volume and good depth of qualitative data supporting the finding. Two studies provided a moderate volume of data [51, 52]. | No/Very minor concerns  **Explanation:** Directly relevant. The finding reflects clinicians’ perspectives on AI without mismatch in population, phenomenon, or setting. | High confidence | There were minor concerns regarding methodology and adequacy. |

| **Summary of review finding** | **Ref** | **Methodological limitations** | **Coherence** | **Adequacy** | **Relevance** | ***CERQual assessment of confidence in the evidence*** | ***Explanation of CERQual assessment*** |
| --- | --- | --- | --- | --- | --- | --- | --- |
| **Data security, privacy and legal implications** | | | | | | | |
| 20. The security of patient data processed by AI is a significant concern for clinicians. | [43, 45-48, 50] | Minor concerns  **Explanation:** The finding is primarily supported by studies with very minor methodological limitations. One study had minor limitations [48]. | Minor concerns  **Explanation:** The finding is supported with high consistency, although there is one contradiction within the finding. | Minor concerns  **Explanation:** Substantial volume and good depth of qualitative data supporting the finding. One study provided a moderate volume of data [43]. | No/Very minor concerns  **Explanation:** Directly relevant. The finding reflects clinicians’ perspectives on AI without mismatch in population, phenomenon, or setting. | High confidence | There were minor concerns regarding methodology, coherence and adequacy. |
| 21. There are significant concerns regarding the legal liability and responsibility when using AI in clinical decisions. | [45, 46, 48, 50, 52, 55] | Minor concerns  **Explanation:** The finding is primarily supported by studies with very minor methodological limitations. One study had minor limitations [48]. | No/Very minor concerns  **Explanation:** High consistency and without significant contradictions throughout the included studies. | Minor concerns  **Explanation:** Substantial volume and good depth of qualitative data supporting the finding. One study provided a moderate volume of data [52]. | No/Very minor concerns  **Explanation:** Directly relevant. The finding reflects clinicians’ perspectives on AI without mismatch in population, phenomenon, or setting. | High confidence | There were minor concerns regarding methodology and adequacy. |

^a^References

**References**

43. Davis M, Dysart GC, Doupnik SK, Hamm ME, Schwartz KTG, George-Milford B, et al. Adolescent, Parent, and Provider Perceptions of a Predictive Algorithm to Identify Adolescent Suicide Risk in Primary Care. Academic Pediatrics. 2024 2024/05/01/;24(4):645-53. doi: https://doi.org/10.1016/j.acap.2023.12.015.

44. Litvin CB, Ornstein SM, Wessell AM, Nemeth LS, Nietert PJ. Adoption of a clinical decision support system to promote judicious use of antibiotics for acute respiratory infections in primary care. International Journal of Medical Informatics. 2012 2012/08/01/;81(8):521-6. doi: https://doi.org/10.1016/j.ijmedinf.2012.03.002.

45. Navarro DF, Kocaballi AB, Dras M, Berkovsky S. Collaboration, not Confrontation: Understanding General Practitioners’ Attitudes Towards Natural Language and Text Automation in Clinical Practice. ACM Transactions on Computer-Human Interaction. 2023;30(2):Article 29. doi: 10.1145/3569893.

46. Kocaballi AB, Ijaz K, Laranjo L, Quiroz JC, Rezazadegan D, Tong HL, et al. Envisioning an artificial intelligence documentation assistant for future primary care consultations: A co-design study with general practitioners. J Am Med Inform Assoc. 2020 Nov 1;27(11):1695-704. PMID: 32845984. doi: 10.1093/jamia/ocaa131.

47. Shibl R, Lawley M, Debuse J. Factors influencing decision support system acceptance. Decision Support Systems. 2013 2013/01/01/;54(2):953-61. doi: https://doi.org/10.1016/j.dss.2012.09.018.

48. Buck C, Doctor E, Hennrich J, Jöhnk J, Eymann T. General Practitioners' Attitudes Toward Artificial Intelligence-Enabled Systems: Interview Study. J Med Internet Res. 2022 Jan 27;24(1):e28916. PMID: 35084342. doi: 10.2196/28916.

49. Ahearn MD, Kerr SJ. General practitioners' perceptions of the pharmaceutical decision-support tools in their prescribing software. Med J Aust. 2003 Jul 7;179(1):34-7. PMID: 12831382. doi: 10.5694/j.1326-5377.2003.tb05415.x.

50. Allen MR, Webb S, Mandvi A, Frieden M, Tai-Seale M, Kallenberg G. Navigating the doctor-patient-AI relationship - a mixed-methods study of physician attitudes toward artificial intelligence in primary care. BMC Primary Care. 2024 Jan 27;25(1):42. PMID: 38281026. doi: 10.1186/s12875-024-02282-y.

51. Nash DM, Thorpe C, Brown JB, Kueper JK, Rayner J, Lizotte DJ, et al. Perceptions of Artificial Intelligence Use in Primary Care: A Qualitative Study with Providers and Staff of Ontario Community Health Centres. The Journal of the American Board of Family Medicine. 2023;36(2):221-8. doi: 10.3122/jabfm.2022.220177R2.

52. Upshaw TL, Craig-Neil A, Macklin J, Gray CS, Chan TCY, Gibson J, et al. Priorities for Artificial Intelligence Applications in Primary Care: A Canadian Deliberative Dialogue with Patients, Providers, and Health System Leaders. The Journal of the American Board of Family Medicine. 2023;36(2):210-20. doi: 10.3122/jabfm.2022.220171R1.

53. Libon J, Ng C, Bailey A, Hareendranathan A, Joseph R, Dulai S. Remote diagnostic imaging using artificial intelligence for diagnosing hip dysplasia in infants: Results from a mixed-methods feasibility pilot study. Paediatrics & Child Health. 2023;28(5):285-90. doi: 10.1093/pch/pxad013.

54. Sangers TE, Wakkee M, Moolenburgh FJ, Nijsten T, Lugtenberg M. Towards successful implementation of artificial intelligence in skin cancer care: a qualitative study exploring the views of dermatologists and general practitioners. Archives of Dermatological Research. 2023 2023/07/01;315(5):1187-95. doi: 10.1007/s00403-022-02492-3.

55. Helenason J, Ekström C, Falk M, Papachristou P. Exploring the feasibility of an artificial intelligence based clinical decision support system for cutaneous melanoma detection in primary care – a mixed method study. Scandinavian Journal of Primary Health Care. 2024 2024/01/02;42(1):51-60. doi:
